# Quality of Life and Functional Outcomes After Rectal Cancer Surgery: A Comparative Study Applying EORTC QLQ-C30, QLQ-CR29, and LARS Score at 1–6 Months Postoperatively

**DOI:** 10.3390/healthcare14091203

**Published:** 2026-04-30

**Authors:** Floris Cristian Stanculea, Claudiu O. Ungureanu, Octav Ginghina, Razvan A. Stoica, Raul Mihailov, Valerii Lutenco, Valentin T. Grigorean, Mircea Litescu, Niculae Iordache

**Affiliations:** 1 General Medicine Faculty, Carol Davila University of Medicine and Pharmacy, 050474 Bucharest, Romania; floris-cristian.stanculea@drd.umfcd.ro (F.C.S.); octav.ginghina@umfcd.ro (O.G.); valentin.grigorean@umfcd.ro (V.T.G.); mircea.litescu@umfcd.ro (M.L.); niordache@gmail.com (N.I.); 2 Department of General Surgery, Sf. Ioan Clinical Hospital of Emergency, 042122 Bucharest, Romania; 3 Department of General Surgery, Prof. Dr. Al. Trestioreanu Oncological Institute, 022328 Bucharest, Romania; 4 General Surgery Department, “Sf. Andrei” Clinical Emergency Hospital, 177 Braila Street, 800578 Galati, Romania; raulmihailov@yahoo.com (R.M.); valerii.lutenco@ugal.ro (V.L.); 5 Department of General Surgery, Bagdasar-Arseni Clinical Emergency Hospital, 041915 Bucharest, Romania; 6 The Academy of Romanian Scientists, 050044 Bucharest, Romania

**Keywords:** rectal cancer, LARS, quality of life, PROM, colostomy, EORTC QLQ-C30, EORTC QLQ-CR29, postoperative outcomes

## Abstract

Background/Objectives: Quality of life (QoL) and functional recovery are essential outcomes in patients undergoing rectal cancer surgery. In addition to oncological results, bowel dysfunction and stoma-related issues may significantly affect postoperative well-being. We aimed to evaluate QoL changes at 1 and 6 months postoperatively and functional outcomes in rectal cancer patients who underwent curative surgical treatment, sphincter-preserving surgeries (SPS) or abdominoperineal resection (APR). Owing to its impact on QoL, several functions were assessed using the Low Anterior Resection Syndrome (LARS) score. Methods: This retrospective observational study consisted of 99 patients who underwent curative rectal cancer surgery, of which 38 patients had colostomy, and 61 no colostomy. To assess patient-reported outcomes related to QoL, the EORTC QLQ-C30 questionnaire, QLQ-CR29 questionnaire, and LARS instrument were sent to the patients at 1 and 6 months postoperatively. Changes over time were analyzed using paired statistical tests, and subgroup analyses were performed according to colostomy status and surgical approach. Results: Significant improvements were observed in the global health status and all major functional domains between 1 and 6 months postoperatively. The global health status increased from 74.9% to 86.5% (*p* < 0.001). Symptom burden decreased significantly, particularly for fatigue (−18.31), pain (−14.48), diarrhea (−12.46), and insomnia (−11.45), representing clinically meaningful improvements. Patients who underwent abdominoperineal resection or resection with colostomy had lower QoL scores at 1 month but showed substantial improvement at 6 months, becoming comparable to those who underwent SPS. LARS outcomes demonstrated progressive functional recovery, with the proportion of patients without LARS increasing from 39 to 46, while major LARS decreased from 7 to 3 patients. However, approximately 40% of patients in the SPS group continued to report moderate-to-severe LARS symptoms. Conclusions: In this study, QoL and bowel function improved significantly during the first 6 months after colorectal cancer surgery. Although most patients demonstrated recovery, persistent bowel dysfunction and stoma-related challenges remain important issues. These findings highlight the need for comprehensive postoperative care and routine assessment of both QoL and functional outcomes.

## 1. Introduction

The incidence of colorectal cancer (CRC) has steadily increased over the past few decades and it is currently the third most commonly diagnosed noncommunicable disease and the fourth leading cause of death globally [1]. Rectal cancer remains a significant global health concern, accounting for approximately one-third of colorectal malignancies. Advances in diagnosis, staging, and multimodal therapy have contributed to improved outcomes; however, the management of rectal cancer continues to present complex challenges, particularly in balancing oncological control with functional preservation. Rectal cancer continues to pose a significant challenge in contemporary surgical oncology, both in terms of achieving oncological clearance and preserving the QoL of patients.

A cancer diagnosis often brings about significant stress, heightened emotional tension, and uncertainty regarding long-term outcomes. These factors are commonly linked to a decline in QoL, particularly in terms of biopsychosocial functioning. Patients are confronted with new adaptive challenges, including altered body image, decreased physical fitness, and difficulties fulfilling social roles. Psychological distress, including stress, anxiety, and depression, is highly prevalent among patients with cancer and is increasingly recognized as a common and expected response to both diagnosis and treatment rather than an atypical reaction [2].

Among various malignancies, colorectal cancer is particularly impactful on mental health and is notably associated with profound changes in the physical self [3]. While multimodal treatment strategies, including chemotherapy and radiotherapy, play an integral role in comprehensive cancer care, surgical resection remains the cornerstone of curative-intent therapy. This approach is associated with 5-year survival rates of 61.6% to 70.9% [4].

Among the most commonly utilized surgical approaches are abdominoperineal resection (APR), low anterior resection (LAR), high anterior resection (HAR), and intersphincter resection (ISR), each selected based on tumor location, stage, and patient-specific factors. Ongoing refinements in surgical technique and perioperative care aim to optimize both oncologic outcomes and postoperative function. 

While APR necessitates a permanent colostomy and is generally reserved for low-lying tumors not amenable to sphincter preservation, Resection with anastomosis aims to maintain sphincter function but may result in complications such as low anterior resection syndrome (LARS), which includes fecal urgency, incontinence, and frequent bowel movements.

Thus, the assumption that sphincter preservation uniformly leads to better QoL has been increasingly challenged. Approximately 41% of patients who undergo sphincter-preserving surgery for rectal cancer without the creation of a stoma experience major LARS one year postoperatively [5]. LARS has been shown to negatively impact both short-term and long-term health-related QoL [6,7].

The challenges faced by patients who undergo surgery resulting in an intestinal stoma—such as stoma management, skin complications, and negative body image—differ from those experienced by patients with preserved gastrointestinal continuity, who commonly report issues such as defecation and urination disorders, sexual dysfunction, and urgency [8,9]. If these symptoms are not alleviated to an acceptable level within a reasonable timeframe, patients may become more vulnerable to developing mental health disorders such as anxiety or depression, as well as experiencing negative long-term effects on health-related QoL [10]. Various factors can adversely affect functional outcomes after rectal surgery, including low-lying anastomoses, the presence of a temporary or preoperative stoma, and the use of (neo)adjuvant radiotherapy. In some cases, opting for a permanent stoma may help avoid these functional impairments. However, this approach also carries its own risks, such as complications related to the stoma itself—including parastomal hernia, prolapse, retraction, and tissue necrosis [11,12]. Temporary stomas are also associated with a notable increase in medium- to long-term complications, often leading to hospital readmissions and the need for additional procedures. Moreover, as many as 28.5% of these stomas are never reversed, becoming permanent [13].

Despite advances in surgical techniques, including total mesorectal excision (TME), which have improved oncological outcomes and enabled greater rates of sphincter preservation, the impact of different surgical strategies on the early postoperative QoL remains a subject of ongoing debate [14,15,16,17]. Anterior resection has become a viable option for low rectal cancer as improved tolerance for shorter distal resection margins, the adoption of TME, and the introduction of circular stapling devices have collectively led to a significant reduction in APR rates [15,16,17]. In addition to oncologic outcomes, sphincter preservation is regarded as an indicator of surgical quality in patients with rectal cancer [18]. Importantly, while many studies have evaluated long-term outcomes, data on early postoperative recovery, particularly within the first six months, remain limited, especially regarding the dynamic changes in QoL and functional adaptation between patients with and without colostomy. Postoperative complications, unsatisfactory functional outcomes, and stoma creation can negatively affect the QoL after rectal surgery. These factors contribute to the complexity of deciding between stoma creation and anastomosis [13]. This choice should be made collaboratively with the patient, as their preferences and values are essential to the decision-making process. Providing clear information about the QoL following rectal cancer surgery is crucial to support informed, shared decision-making [19]. Therefore, the primary aim of this study was to evaluate the changes in QoL and functional outcomes at 1 and 6 months following rectal cancer surgery. The secondary objectives included comparing outcomes between patients with and without colostomy and assessing bowel dysfunction using the LARS score in patients undergoing sphincter-preserving procedures. The study cohort included 99 patients with rectal cancer, with a mean age of 67.5 years and a predominance of male patients (62.6%). Most patients presented with stage II–III disease, whereas a smaller proportion had stage I tumors. The detailed demographic and clinical characteristics are presented in Table 1.

## 2. Materials and Methods

A retrospective cohort study was conducted involving 99 patients diagnosed with rectal cancer who underwent curative surgical treatment at St. John Emergency Hospital, Surgical Oncology Department, Bucharest, Romania between January 2023 and September 2025. Ethical approval has been granted by the Ethics Committee from St. John Emergency Hospital (reference: No 16724. December 2022). Written informed consent for participation in the study, as well as the collection of their clinical data, was obtained from all patients. The questionnaires were provided to patients during scheduled clinical appointments or administered remotely via telephone.

Patients were divided into two groups based on the type of surgical intervention: 38 patients underwent abdominoperineal resection and resection with colostomy, and 61 underwent LAR, HAR and ISR. First group—colostomy and second group no colostomy. All surgeries were performed by a high-volume surgeon (>50 colorectal procedures per year). All surgical interventions were performed no sooner than 12 weeks after the initiation of radiotherapy and at least 4 weeks after the completion of chemotherapy, and were only performed in patients with remaining tumor tissue. Surgery was not performed in cases in which a complete response was observed. Adjuvant therapy, either chemotherapy or radiotherapy, was administered based on the pathological findings. Patients with more advanced locoregional disease received adjuvant treatment for up to six months after the initial therapy.

Performance status (PS) of a patient is the assessment of level of function and capability of self-care and an important factor in determining QoL. A commonly used tool is The Eastern Cooperative Oncology Group (ECOG) performance status [20]. We used ECOG score to determine the eligibility of patients with good overall health status.

Baseline characteristics were comparable between groups in terms of eligibility criteria (ECOG 0–1, non-metastatic disease), although no formal matching or statistical adjustment was performed.

Inclusion criteria were:

- Written consent to participate in the study;
- Good overall health status (ECOG of 0–1);
- Histologically confirmed rectal adenocarcinoma;
- Elective surgical resection;
- Availability for postoperative follow-up for a minimum of 6 months;
- No distant metastases at the time of surgery or local advanced cancer;
- Clinical stage I–III (pTNM/ypTNM).

Exclusion criteria included:

- Recurrent disease;
- Incomplete data or loss to follow-up;
- Pre-existing severe psychiatric or neurological disorders impacting QoL evaluation;
- Serious medical conditions that elevate the risk of perioperative complications;
- Pre-existing altered conditions of anal sphincter complex;
- Survival of less than 12 months;
- Temporary ileostomy protection.

### 2.1. Examination Protocol

Patient-reported outcomes (PROs) provide reports from patients about their own health, QoL, or functional status related to health care or treatment they have received. Patient-reported outcomes measures (PROMs) are tools and/or instruments used to capture these PROs [21].

To assess PROs, we initiated an examination protocol consisting of the following steps:

- Obtaining informed consent;
- Collecting patient history, including demographic, social, and clinical details (such as procedure eligibility, neoadjuvant or adjuvant treatments, cancer staging, and duration of hospital stay);
- Completion of the QLQ-C30,QLQ-C29 (validated questionnaires by the European Organization for Research and Treatment of Cancer) and LARS questionnaires to assess QoL.

All questionnaires were administered during scheduled postoperative clinical visits or via telephone interviews conducted by trained personnel. Assessments were performed approximately 1 month (±1 week) and 6 months (±1 week) postoperatively. Only patients who completed all questionnaires at both time points were included in the final analysis, and cases with incomplete data were excluded to ensure the consistency of longitudinal comparisons.

It should be noted that the selection of patients included in the study may also influence PROs, as patients with better baseline functional status or fewer comorbidities may experience more favorable recovery. The questionnaires used in this study (EORTC QLQ-C30, QLQ-CR29, and LARS) are validated instruments and are provided as Supplementary Material.

### 2.2. Objectives

This study aims to provide a comprehensive evaluation of postoperative recovery by integrating QoL assessment with functional bowel outcomes, including LARS, and comparing patients with and without colostomy during the early postoperative period.

The primary endpoint of the study is to compare the QoL in patients with rectal cancer who have APR or resection with colostomy versus those treated with LAR, HAR or ISR.

The study also aimed to assess the prevalence of low anterior resection syndrome and the psychosocial implications of permanent stomas. We also evaluated functional bowel outcomes, psychological well-being, and overall patient satisfaction post-surgery.

### 2.3. Quality of Life (QoL)

One class of PRO measures health-related quality of life (HRQL). The concept of HRQL was first introduced by Schipper and encompasses four key dimensions: physical (motor), psychological, social and economic, and somatic well-being. It emphasizes an individual’s subjective perception of their health status, including symptoms and the impact of disease-related complications. Building on this framework, the idea was refined by distinguishing between objectively measurable health conditions and the patient’s personal experience of symptoms [22]. Evaluating QoL provides surgeons with valuable insights into the full range of outcomes following a procedure. According to O’Boyle, surgical success should not be measured solely by mortality rates, complication rates, or clinical outcomes. The patient’s overall satisfaction, clearly reflected in their post-operative QoL, is an essential factor in assessing the true effectiveness of the intervention [23]. Several studies have investigated the impact of surgical choices on long-term QoL in patients with rectal cancer, various strategies and efforts to improve QoL outcomes [24]. While HAR, LAR and ISR offer the advantage of sphincter preservation, they are not without complications. LARS, affecting up to 80% of patients post-LAR, includes symptoms such as incontinence, urgency, and fragmentation of bowel movements, which can significantly impair daily functioning and social engagement.

In contrast, APR or resection with colostomy, although resulting in a permanent or temporary colostomy, often leads to more predictable bowel function and fewer instances of urgency or incontinence. Research has shown that patients with well-managed stomas may report equal or even superior QoL compared to those experiencing severe LARS. Psychological factors, body image, and social reintegration are key determinants in both groups and must be considered in any comprehensive evaluation of QoL outcomes.

QoL is typically assessed using validated instruments, such as the EORTC QLQ-C30, QLQ-CR29, or LARS score, providing structured insights into physical, emotional, and social well-being. However, there remains variability in how QoL is interpreted and reported across studies, underlining the need for more consistent and PROMs.

#### 2.3.1. QoL Measurement Tools

QoL was assessed using validated tools: the EORTC QLQ-C30 and colorectal cancer-specific module QLQ-CR29 questionnaires at two time points: during the immediate postoperative period (within 1 month) and at 6 months postoperatively. Scores were evaluated across domains including physical function, role function, emotional well-being, gastrointestinal symptoms, and body image. Additionally, the LARS score was applied to patients in the LAR, HAR and ISR (no colostomy) group to assess bowel function.

##### The EORTC QLQ-C30 Questionnaire

The EORTC QLQ-C30 is a validated questionnaire designed for self-assessment of QoL in cancer patients, regardless of tumor location or stage. It includes five functional scales that evaluate physical, emotional, cognitive, and social functioning, as well as role performance. Additionally, it features three symptom scales measuring fatigue, pain, and nausea/vomiting, along with a global health status/QoL scale for overall self-evaluation. The questionnaire also addresses six individual symptoms: loss of appetite, diarrhea, constipation, sleep disturbances, dyspnea, and financial difficulties related to the illness.

##### The QLQ-CR29 Questionnaire

The QLQ-CR29 is specifically designed to evaluate symptoms in patients with colorectal cancer. This module includes 29 questions addressing various health issues commonly encountered during and after treatment.

All raw scores were linearly transformed to a 0–100 scale according to the EORTC scoring manual. In both the QLQ-C30 and QLQ-CR29, higher scores on functional scales indicate better functioning in specific areas. In contrast, higher scores on symptom scales reflect greater symptom severity and a corresponding decline in QoL [25,26,27]. Differences between time points were evaluated using paired statistical tests, and statistical significance was defined as *p* < 0.05. In addition to statistical significance, clinical relevance was interpreted according to established EORTC guidelines, where changes of 5–10 points represent small differences, 10–20 points moderate differences, and >20 points large clinically meaningful changes [26,27,28].

#### 2.3.2. LARS Score

LARS, first described in 2012, represents a group of bowel dysfunction symptoms that may occur following rectal cancer surgery [5]. These symptoms commonly include urgency, fecal incontinence, increased stool frequency with clustering, and a sensation of incomplete evacuation [5].

LARS has been shown to negatively affect patients’ QoL. It is estimated that up to 75% of patients experience LARS symptoms within the first postoperative year, with a substantial proportion continuing to report symptoms even after long-term follow-up. Given both the high prevalence and persistence of these symptoms, a better understanding of the natural course of LARS and the development of effective management strategies remains important clinical priority [29,30]. To date, there is no consensus as to the optimal treatment strategy for LARS [31].

### 2.4. Statistical Analysis

Continuous variables are expressed as mean ± standard deviation, and data distribution was assessed using the Shapiro–Wilk test. Changes in QoL scores between the two postoperative time points (1 and 6 months) were analyzed using paired sample *t*-tests or the Wilcoxon signed-rank test, depending on the data distribution. Between-group comparisons were performed using independent sample *t*-tests or the Mann–Whitney U test as appropriate. Categorical variables were analyzed using the chi-square test. Mean differences (Δ) were calculated to assess the magnitude of the changes over time. Given the exploratory nature of the study and the number of outcomes assessed, no formal adjustments for multiple comparisons were performed. Statistical significance was set at *p* < 0.05.

## 3. Results

### 3.1. Results of EORTC QLQ-C30

**Figure 1:**
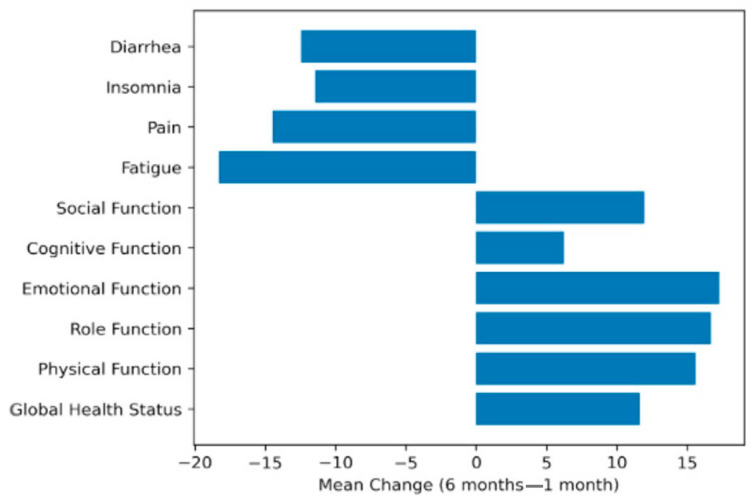
Changes in QoL scores between 1 and 6 months postoperatively measured using the EORTC QLQ-C30. Positive values indicate improvement in functional scales and global health status, while negative values represent reductions in symptom burden.

**Figure 2:**
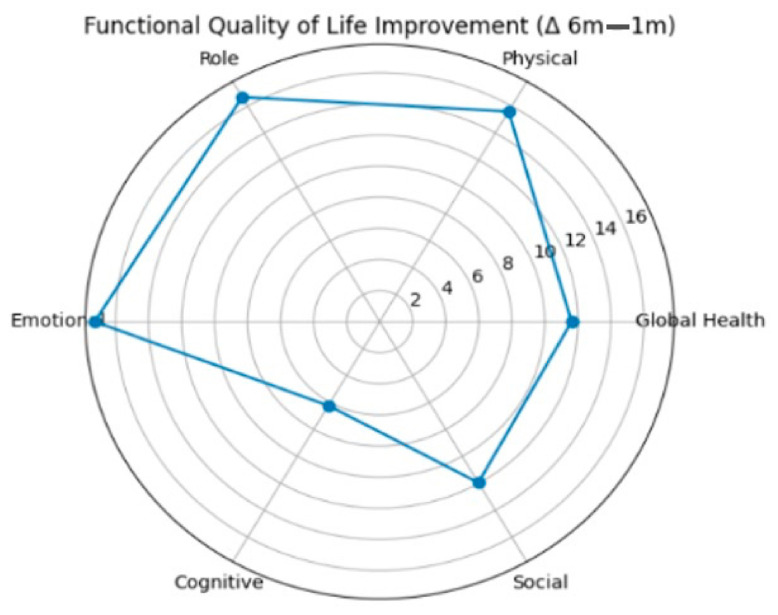
Radar plot illustrates changes in functional QoL domains between 1 and 6 months postoperatively measured using the EORTC QLQ-C30. Positive values indicate improvement in patient-reported outcomes.

All included patients (n = 99) completed the assessments at both time points, and no missing data were present in the analyzed cohort (Table 2). A significant improvement was observed in the global health status between 1 and 6 months postoperatively (mean score 74.92 vs. 86.53; Δ = +11.62, *p* < 0.001). Similarly, all functional scales demonstrated statistically significant improvements over time, including physical functioning (70.89 vs. 86.50; Δ = +15.61, *p* < 0.001), role functioning (67.35 vs. 84.01; Δ = +16.67, *p* < 0.001), emotional functioning (68.04 vs. 85.31; Δ = +17.27, *p* < 0.001), cognitive functioning (89.06 vs. 95.29; Δ = +6.23, *p* < 0.001), and social functioning (78.28 vs. 90.24; Δ = +11.95, *p* < 0.001). Regarding symptom scales, significant reductions were observed for fatigue (29.73 vs. 11.42; Δ = −18.31, *p* < 0.001), pain (18.01 vs. 3.54; Δ = −14.48, *p* < 0.001), insomnia (22.56 vs. 11.11; Δ = −11.45, *p* < 0.001), diarrhea (21.55 vs. 9.09; Δ = −12.46, *p* < 0.001), and financial difficulties (21.21 vs. 12.12; Δ = −9.09, *p* < 0.001). Smaller but statistically significant improvements were also noted for nausea and vomiting, dyspnea, appetite loss, constipation, and urinary symptoms (Figure 1 and Figure 2).

### 3.2. Results of EORTC QLQ-CR29

Analysis of the QLQ-CR29 module revealed significant decreases in colorectal cancer-specific symptoms postoperatively. Improvements were observed in abdominal pain, buttock pain, bloating, stool frequency, flatulence, and fecal incontinence (*p* < 0.001 for most comparisons). Stoma-related problems also significantly decreased among patients with a colostomy (n = 38). Psychosocial outcomes improved as well, with a marked reduction in anxiety scores and improved body image perception Sexual interest showed a slight increase in both men and women; however, these changes did not reach statistical significance. Detailed results are presented in Table 3.

QoL analysis demonstrated significant improvements across most domains between 1 and 6 months postoperatively. Global health status and all functional scales showed statistically and clinically meaningful increases, particularly in emotional and role functioning. In parallel, several symptom domains, including fatigue, pain, insomnia, and gastrointestinal symptoms, showed significant reductions over time. According to established EORTC interpretation guidelines, many of these changes exceeded the threshold for moderate clinical significance (>10 points), suggesting that postoperative recovery was associated with substantial improvements in patient-reported outcomes. Furthermore, colorectal cancer-specific symptoms assessed by the QLQ-CR29 module, including abdominal pain, bloating, and stool-related symptoms, also improved significantly. Stoma-related difficulties decreased considerably among patients with a colostomy, indicating adaptation over time.

### 3.3. Clinical Meaning of the Relevant Features of QLQ-CR29 and QLQ-C30

QoL analysis demonstrated clinically meaningful improvements across most domains between 1 and 6 months postoperatively (interpretation key in Table 4). Global health status and functional scales showed moderate improvements, particularly in emotional and role functioning scores. Symptom burden decreased substantially, with fatigue showing a large improvement and pain and stoma-related problems showing moderate-to-large improvements. Anxiety demonstrated a very large reduction, representing one of the most significant changes. Many of these changes exceeded the threshold for clinically meaningful differences (≥10 points). We detailed these results in Table 5.

**Figure 3:**
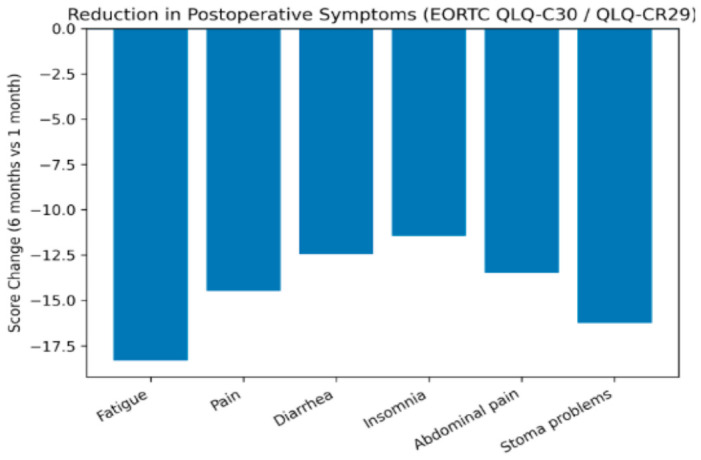
Changes in EORTC QLQ-C30 and QLQ-CR29 symptom scores between 1 and 6 months postoperatively. Negative values indicate symptom improvement.

Disease-specific outcomes measured using the EORTC QLQ-CR29 also demonstrated meaningful improvements. Symptoms related to bowel function, abdominal discomfort, and urinary disturbances decreased significantly over time (Figure 3). Additionally, stoma-related difficulties were significantly reduced among patients with a colostomy, which may reflect improved adaptation and patient education during postoperative follow-up. Importantly, many observed changes exceeded the threshold for clinically meaningful differences defined by EORTC guidelines, suggesting that the improvements were not only statistically significant but also clinically relevant. Emotional functioning and fatigue demonstrated particularly large changes, highlighting the importance of psychosocial recovery during the postoperative period. Despite these improvements, some aspects of QoL, particularly sexual function and sexual interest, showed limited change and did not reach statistical significance.

### 3.4. QoL According to Colostomy Status

QoL outcomes were analyzed between patients with colostomy (n = 38) and those without colostomy (n = 61).

At 1 month post-operation, patients with a colostomy generally reported lower functional scores and higher symptom burden compared to patients without colostomy. Both groups demonstrated significant improvements in most domains by 6 months.

Patients with colostomy exhibited greater improvements over time in several domains, particularly in global health status, physical functioning, and fatigue, despite worse baseline scores (Table 6).

Among patients without colostomy, significant improvements were also observed across the functional domains and symptom scales, including fatigue, pain, insomnia, and diarrhea. Anxiety showed the most pronounced reduction in this group. Patients with colostomy demonstrated greater improvements over time, despite worse baseline scores. At 6 months, no statistically significant difference in global health status was observed between the groups.

#### Stoma-Specific Outcomes

Stoma-specific outcomes assessed using the QLQ-CR29 module also improved significantly over time. Colostomy-related problems decreased from 29.06 to 12.82 (Δ = −16.24, *p* < 0.001), indicating improved adaptation to stoma management during follow-up. Additionally, embarrassment related to bowel function showed a marked reduction (Δ = −23.08, *p* < 0.001), suggesting improved psychosocial adjustment among patients with a stoma.

Despite these improvements, some domains remained relatively impaired among patients with a colostomy, particularly body image and sexual functioning, which showed smaller or non-significant changes during follow-up. Overall, these findings indicate that although patients with a colostomy experience a greater early postoperative burden, substantial improvements occur during the first six months following surgery, with many domains demonstrating clinically meaningful recovery.

### 3.5. LARS Score Results

Functional outcomes assessed using the LARS score were evaluated only in patients without colostomy (n = 61), as patients with a stoma are not evaluable for LARS. Functional outcomes assessed using the LARS score demonstrated a clear trend toward improvement between 1 and 6 months postoperatively. At 1 month, among evaluable patients (n = 61), 39 (63.9%) were classified as having no LARS, 15 as minor LARS, and 7 as major LARS, while 38 patients had a colostomy and were therefore not evaluable for LARS. At 6 months, the proportion of patients without LARS increased to 46, whereas the number of patients with minor and major LARS decreased to 12 and 3, respectively. The number of patients with colostomy remained unchanged.

Analysis of category transitions further highlighted this improvement. Among patients without LARS at 1 month, 36 (92.3%) remained symptom-free at 6 months. Of the 15 patients with minor LARS, 9 (60.0%) improved to no LARS, 5 (33.3%) remained stable, and only 1 (6.7%) progressed to major LARS. Among the 7 patients with major LARS at 1 month, 5 (71.4%) showed improvement by 6 months (1 to no LARS and 4 to minor LARS), while 2 (28.6%) remained in the major LARS category. Most patients showed improvement over time, with only a small proportion experiencing persistent major LARS.

When comparing surgical approaches, patients who underwent APR or resection with colostomy exhibited significantly lower QoL scores in the early postoperative period compared with those who underwent resection with anastomosis (HAR, LAR, ISR). These differences were particularly evident in domains related to body image, social functioning, and emotional well-being (*p* < 0.05). APR patients also reported reduced physical activity levels and increased psychological distress, likely associated with the presence of a permanent colostomy.

**Figure 4:**
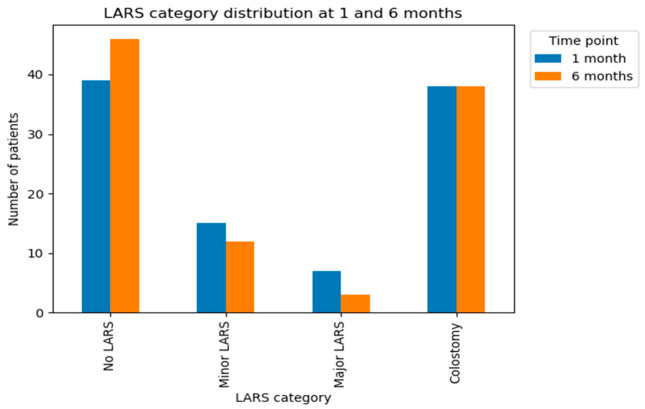
LARS score distribution.

However, at 6 months postoperatively, QoL scores in the colostomy group improved substantially and became comparable to those observed in the anastomosis group across most functional domains (*p* > 0.05), with no statistically significant difference in global health status between the two groups (Figure 4). In contrast, although patients undergoing resection with anastomosis generally reported higher early postoperative QoL scores, approximately 40% continued to experience moderate to severe LARS symptoms, including urgency, incontinence, and stool fragmentation, which persisted at 6 months and negatively impacted daily functioning.

## 4. Discussion

The present study evaluated postoperative changes in QoL among patients undergoing colorectal cancer surgery using the validated instruments EORTC QLQ-C30, the colorectal cancer-specific module EORTC QLQ-CR29, and LARS scores. Our findings demonstrate significant improvements in global health status, functional domains, and symptom burden between one and six months after surgery. These results suggest a progressive recovery in both the physical and psychosocial aspects of QoL during the early postoperative period. These findings are consistent with those of previous studies evaluating QoL after rectal cancer surgery. For example, improvements in global health status and functional domains during the early postoperative period have been reported in recent studies (Jung, 2024) [24]. Several studies have demonstrated that QoL significantly improves during the first postoperative months following colorectal cancer surgery [32,33,34]. Our findings are consistent with recent evidence from a systematic review of randomized controlled trials, which highlights the importance of organ-preserving strategies and structured postoperative care in improving the health-related QoL of patients with rectal cancer [34].

Global health status increased significantly during follow-up, indicating an overall improvement in patients’ perception of their general health and well-being. Similar improvements in global health status have been reported in longitudinal studies evaluating postoperative recovery in patients with colorectal cancer, in which QoL scores typically decline in the early postoperative period and gradually improve during subsequent months [35]. The improvements observed in our study likely reflect the gradual resolution of postoperative symptoms, recovery of physical capacity, and psychological adaptation following surgical treatment.

Improvements in global health status and functional domains observed in this study are consistent with findings reported in previous research using the EORTC QLQ-C30 [25]. For example, studies of rectal cancer survivors have shown that physical and role functioning tend to improve gradually within the first six months after surgery as patients recover from surgical trauma and resume daily activities [36].

Functional outcomes improved significantly across all domains. Physical and role functioning demonstrated marked improvements, suggesting that many patients were able to resume daily activities and regain functional independence within the first six postoperative months. Emotional functioning showed one of the largest improvements among the functional scales, indicating substantial psychological recovery following cancer treatment. Improvements in social functioning further support the concept that patients progressively reintegrate into social and interpersonal activities as postoperative recovery progresses. These findings are consistent with previous studies reporting progressive improvement in functional status after colorectal cancer surgery [37].

Symptom scales also demonstrated substantial improvements during follow-up. Fatigue, pain, insomnia, and gastrointestinal symptoms decreased significantly between the two assessment time points. Fatigue showed one of the largest reductions in symptom burden. This finding is particularly relevant because fatigue represents one of the most frequently reported symptoms among colorectal cancer patients and may significantly affect daily functioning and overall well-being. The observed reduction in pain and insomnia likely reflects both surgical recovery and improvements in overall physical health during the postoperative period. Disease-specific symptoms assessed with the QLQ-CR29 module also improved significantly. Abdominal pain, bloating, stool frequency, and fecal incontinence showed meaningful reductions over time, suggesting gradual recovery of gastrointestinal function following surgical treatment. Improvements in urinary symptoms were also observed, which may reflect progressive recovery of pelvic floor and autonomic nerve function after surgery [38]. Studies using the EORTC QLQ-CR29 instrument have shown that gastrointestinal symptoms such as diarrhea, abdominal pain, and stool frequency are most pronounced during the early postoperative phase but tend to improve over time. These findings are consistent with our results, which demonstrate significant reductions in several gastrointestinal symptoms between one and six months after surgery [36].

Stoma-related outcomes represent an important aspect of postoperative QoL in colorectal cancer patients [38]. In our study, patients with a colostomy initially reported lower QoL scores compared with those without a stoma, particularly in domains related to physical functioning, body image, and social interaction. However, previous research has demonstrated that these differences tend to diminish over time as patients adapt to stoma management and regain functional independence [39,40,41]. Our findings support this observation, as significant improvements were observed in both functional and symptom domains among patients with a colostomy during the first six postoperative months.

An important observation in this study is that several of the changes exceeded the thresholds for clinically meaningful differences defined by the EORTC guidelines. According to established interpretation criteria, changes greater than 10 points are generally considered clinically relevant. In our cohort, improvements exceeding this threshold were observed for multiple domains, including emotional functioning, role functioning, physical functioning, fatigue, and several gastrointestinal symptoms. These results indicate that the improvements observed are not only statistically significant but also clinically meaningful from the patient’s perspective.

The improvements observed in the present study are consistent with previous research evaluating QoL after colorectal cancer surgery. Several longitudinal studies have demonstrated that QoL scores decline during the immediate postoperative period but gradually improve over the following months as patients recover from surgical trauma and adapt to functional changes. For example, Whistance RN et al. reported progressive improvement in global health status and functional domains during the first postoperative year following colorectal cancer surgery, particularly in physical and emotional functioning [32]. Similarly, Lindsetmo RO and Norstein J observed that symptom burden, including fatigue and gastrointestinal complaints, decreases significantly during postoperative recovery [33]. 

The impact of stoma formation on postoperative QoL has also been widely studied. Previous investigations have reported that patients with a permanent stoma initially experience lower QoL scores, particularly in domains related to body image, social functioning, and psychological well-being [34,42]. However, several studies have demonstrated that these differences often diminish during follow-up as patients adapt to stoma care and regain independence [43,44]. Our results support these observations and show significant improvements in both functional status and stoma-related symptoms during the postoperative period; therefore, it is important for these patients to receive appropriate postoperative education and support.

Emotional functioning often improves over time as patients adapt psychologically to their diagnosis and treatment. The symptom improvement observed in this cohort, particularly reductions in fatigue, pain, and gastrointestinal symptoms, is consistent with previously reported postoperative trajectories. Significant reductions in fatigue, pain, anxiety, and insomnia were observed, suggesting that a series of postoperative symptoms diminish as recovery progresses. Anxiety showed very large improvement (−44.11) and fatigue showed large improvement (−18.31). These findings are consistent with previous studies on colorectal cancer survivorship, which report gradual improvement in symptom burden within the first postoperative months [43,44,45].

Sexual dysfunction refers to a set of symptoms such as impotency, inability to ejaculate, erectile dysfunction, lack of sexual desire or dyspareunia and remains one of the most persistent QoL challenges following colorectal cancer surgery [46].

Prior studies have reported that pelvic autonomic nerve injury and psychosocial factors may contribute to long-term sexual dysfunction in both men and women [47,48]. Consistent with these findings, our study observed limited improvement in sexual interest and functioning, highlighting the need for dedicated counseling and supportive care addressing sexual health during survivorship.

In the present study, changes in sexual interest and sexual functioning did not reach statistical significance, which is consistent with previous reports indicating that sexual dysfunction may persist long after surgical treatment [49]. These findings highlight the importance of addressing sexual health during postoperative follow-up and providing appropriate counseling and supportive interventions for patients experiencing such difficulties [46,47].

The longitudinal analysis of LARS categories suggests that postoperative bowel dysfunction following rectal resection is frequently transient and improves over time. The substantial shift from minor and major LARS toward the no LARS category supports the concept of functional adaptation during the early postoperative period. Importantly, only a small proportion of patients demonstrated persistent or worsening symptoms, underscoring the dynamic nature of postoperative functional outcomes. The temporal pattern of functional recovery observed in our cohort aligns closely with trends reported in recent prospective studies.

In a longitudinal analysis of patients undergoing total mesorectal excision for rectal cancer, the prevalence of major LARS was highest at 1 month and declined by 6 months, with the greatest proportion of spontaneous improvement occurring within the first six postoperative months [49,50]. Similar to our findings, this study reported that the majority of transitions toward less severe LARS categories take place early in the recovery process, with relative stabilization beyond 6 months [31].

Another longitudinal investigation also demonstrated significant reductions in LARS severity up to 6 months after surgery, although persistent symptoms continued to affect a substantial proportion of patients thereafter [51].

Incidence rates of LARS reported in the wider literature vary, with some large observational cohorts indicating that up to 40–50% of patients experience major LARS at longer-term follow-up, underscoring the substantial burden of postoperative bowel dysfunction following sphincter-preserving surgery [52].

The variability in reported incidence highlights the influence of patient, tumor, and treatment-related factors—including neoadjuvant therapy, tumor height, and stoma management—on functional outcomes [31,52,53]. Compared with these broader cohorts, our results emphasize a similar pattern of early functional improvement, while also illustrating that complete resolution of major LARS is relatively uncommon within the first six months. Previous studies have shown that major LARS and chronic pain are associated with significantly impaired functioning and increased symptom burden, negatively impacting the health-related QoL Slørdahl et al., 2024) [54]. These findings should be interpreted with caution, as the observed differences between the groups may be influenced by baseline characteristics and treatment selection rather than the surgical approach alone. However, data specifically addressing early postoperative QoL changes within the first six months remain limited, particularly in cohorts comparing patients with and without colostomy. This highlights an important gap in the literature and supports the relevance of the present study.

### Limitations

Several limitations of this study should be acknowledged. First, the study represents a single-center experience and the sample size was relatively modest, which may limit the generalizability of the findings. Second, QoL assessment was limited to two postoperative time points. Although this design allowed evaluation of short-term recovery, longer follow-up would provide additional insight into long-term QoL trajectories. Third, potential selection bias should be considered, as only patients meeting strict inclusion criteria (ECOG 0–1, non-metastatic disease, and completed follow-up) were included, which may limit the cohort’s representativeness. Additionally, survivorship bias may have been present, as patients with incomplete follow-up or poor outcomes may have been excluded from the analysis. Furthermore, heterogeneity between groups, including differences in baseline characteristics or disease severity, may have influenced the observed outcomes, and the results should not be attributed solely to surgical approaches. Finally, additional analyses evaluating predictors of postoperative QoL improvement, such as age, tumor stage, surgical approach, and adjuvant therapy, may further clarify factors influencing patient recovery. Therefore, future research should seek to validate these findings through multicentre studies or external validation cohorts to confirm the generalisability of the results. Additionally, a further limitation lies in the subjective nature of patient-reported QoL assessments, which may be influenced by psychological, social, and contextual factors. It should be noted that comorbidity burden and chemotherapy regimens were not included in the multivariable analysis. This was due to the absence of the inconsistent collection of comorbidity information which was not expected to introduce substantial variability. Nevertheless, residual confounding cannot be entirely excluded. Additionally, no multivariate analysis was performed to adjust for potential confounders between the surgical groups, which may limit the causal interpretation of the findings.

Despite these limitations, this study provides valuable information regarding the evolution of QoL after colorectal cancer surgery. The use of validated patient-reported outcome instruments and the comprehensive evaluation of both functional and symptom domains allow a detailed assessment of postoperative recovery. Continuous monitoring of QoL outcomes may help clinicians identify patients at risk of persistent symptoms and guide targeted supportive interventions aimed at improving long-term survivorship outcomes.

## 5. Conclusions

The results of this study indicate that QoL improves substantially during the first six months after surgery, with significant gains in functional status and reductions in symptom burden. Patients with colostomy appear to achieve comparable QoL outcomes to those undergoing sphincter-preserving procedures over time, despite worse early postoperative scores. Postoperative recovery in patients with colorectal cancer improves significantly during the first six months after surgery. Functional recovery and symptom reduction occur progressively, reflecting both physical healing and psychological adaptation. However, certain issues, including sexual dysfunction, body image concerns, and stoma-related difficulties may persist and require targeted multidisciplinary interventions. A substantial proportion of patients who undergo sphincter-preserving surgery continue to experience bowel dysfunction, as reflected by persistent LARS symptoms. Therefore, continuous assessment of PROMs should be integrated into postoperative follow-ups to optimize long-term patient care and survivorship. These findings underscore the importance of structured postoperative follow-up and supportive care aimed at optimizing recovery and patient well-being. These findings should be interpreted with caution, as the observed differences between groups may be influenced by baseline characteristics and treatment selection rather than the surgical approach alone. Future studies with longer follow-up periods and multicenter cohorts are needed to better understand the long-term evolution of postoperative QoL.

### Future Directions

An alternative application of brief QoL instruments is their incorporation into routine follow-up visits, particularly in resource-constrained settings. In such contexts, patients could complete the questionnaire while waiting for their consultation. The responses may then be processed using digital scoring systems or automated alerts that flag specific concerns, such as fatigue or gastrointestinal symptoms. This enables clinicians to rapidly identify and prioritise relevant issues, thereby supporting a more focused and efficient consultation. In addition, repeated administration of these questionnaires facilitates longitudinal monitoring, as declines in QoL may indicate clinical deterioration. Overall, in low-resource environments, these cost-effective and time-efficient tools can enhance patient care without requiring additional personnel or infrastructure.

## Figures and Tables

**Table 1:** Demographic and clinical characteristics.

Variable	Total (n = 99)	Colostomy (n = 38)	No Colostomy (n = 61)
Age, years (mean ± SD)	68.7 years	70.7 years	67.45 years
Sex, n (%)			
Male	62	21	41
Female	37	17	20
Tumor stage, n (%)			
Stage I	16	3	13
Stage II	25	10	15
Stage III	58	25	33

**Table 2:** QLQ-C30 symptoms.

Scale	n	1 Month Mean	6 Months Mean	Mean Change (Δ)	*p*-Value
Global health status	99	74.92	86.53	+11.62	<0.001
Functional scales (QLQ-C30)					
Physical functioning	99	70.89	86.50	+15.61	<0.001
Role functioning	99	67.35	84.01	+16.67	<0.001
Emotional functioning	97	68.04	85.31	+17.27	<0.001
Cognitive functioning	99	89.06	95.29	+6.23	<0.001
Social functioning	99	78.28	90.24	+11.95	<0.001
Symptom scales (QLQ-C30)					
Fatigue	99	29.73	11.42	−18.31	<0.001
Nausea/vomiting	99	8.92	2.36	−6.57	<0.001
Pain	99	18.01	3.54	−14.48	<0.001
Dyspnea	99	8.59	3.03	−5.56	<0.001
Insomnia	99	22.56	11.11	−11.45	<0.001
Appetite loss	99	12.79	5.05	−7.74	<0.001
Constipation	99	8.75	4.71	−4.04	0.041
Diarrhea	99	21.55	9.09	−12.46	<0.001
Financial difficulties	99	21.21	12.12	−9.09	<0.001

Detailed results are presented in Table 1.

**Table 3:** QLQ-CR29 Selected Symptoms.

Scale	n	1 Month	6 Months	Δ	*p*
Urinary frequency	99	28.11	15.32	−12.79	<0.001
Abdominal pain	99	15.15	1.68	−13.47	<0.001
Buttock pain	99	18.18	5.72	−12.46	<0.001
Bloating	99	17.17	5.72	−11.45	<0.001
Flatulence	99	24.91	14.48	−10.44	<0.001
Fecal incontinence	99	17.17	8.42	−8.75	<0.001
Stool frequency	99	20.71	9.76	−10.94	<0.001
Stoma problems	39	29.06	12.82	−16.24	<0.001

**Table 4:** Interpretation key for QLQ-CR29/QLQ-C30.

Score Difference	Interpretation
5–10	small change
10–20	moderate clinically meaningful change
>20	large clinical improvement/deterioration

**Table 5:** QLQ-CR29 and QLQ-C30 clinical meaning.

Scale	Δ	Clinical Meaning
1. Global health status	+11.62	moderate improvement
2. Physical functioning	+15.61	moderate improvement
3. Role functioning	+16.67	moderate improvement
4. Emotional functioning	+17.27	moderate improvement
5. Fatigue	−18.31	large improvement
6. Pain	−14.48	moderate improvement
7. Stoma problems	−16.24	moderate improvement
8. Anxiety	−44.11	very large improvement

**Table 6:** Comparison between the two groups: colostomy versus no colostomy.

Domain	No Colostomy Δ (1–6 Month)	Colostomy Δ (1–6 Month)	Clinical Meaning
Global Health Status	+8.75	+16.02	Moderate improvement in both
Physical Function	+14.46	+17.35	Moderate improvement
Role Function	+14.97	+19.23	Moderate improvement
Emotional Function	+17.54	+16.84	Moderate improvement
Cognitive Function	+5.83	+6.84	Small improvement
Social Function	+11.11	+13.25	Moderate improvement
Fatigue	−15.28	−22.96	Large improvement
Pain	−12.78	−17.09	Moderate–large improvement
Abdominal Pain	−10.56	−17.95	Moderate–large improvement
Buttock Pain	−8.33	−18.80	Moderate–large improvement
Diarrhea	−15.00	−8.55	Moderate improvement
Urinary Frequency	−11.39	−14.96	Moderate improvement
Anxiety	−47.22	−39.32	Very large improvement
Body Image	−12.22	−14.87	Moderate improvement
Stoma Problems	N/A	−16.24	Moderate improvement
Embarrassment	−5.00	−23.08	Moderate vs. large improvement

## Data Availability

The data in unavailable due to privacy, but can be provided anytime.

## Supplementary Materials

The following supporting information can be downloaded at: https://www.mdpi.com/article/10.3390/healthcare14091203/s1, File S1: EORTC QLQ-CR29; File S2: SCORE LARS; File S3: EORTC QLQ-C30.

## Abbreviations

The following abbreviations are used in this manuscript:

SPS	sphincter
preserving surgeries	
CRC	colorectal cancer
LAR	low anterior resection
APR	abdominoperineal resection
HAR	high anterior resection
ISR	intersphincter resection
LARS	Low anterior resection syndrome
DRM	distal resection margins
TME	mesorectal excision
HRQL	Health
Related Quality of Life	
QoL	Quality of life
PRO	patient reported outcome
PROM	patient reported outcome measure

## References

[1] Ferlay J., Autier P., Boniol M., Heanue M., Colombet M., Boyle P. (2007). Estimates of the cancer incidence and mortality in Europe in 2006. Ann. Oncol..

[2] Getie A., Edmealem A., Kitaw T.A. (2025). Comparative Impact of Integrated Palliative Care vs. Standard Care on the Quality of Life in Cancer Patients: A Global Systematic Review and Meta-Analysis of Randomized Controlled Trials. PLoS ONE.

[3] Lange M.M., Rutten H.J., van de Velde C.J.H. (2009). One hundred years of curative surgery for rectal cancer: 1908–2008. Eur. J. Surg. Oncol..

[4] Araghi M., Arnold M., Rutherford M.J., Guren M.G., Cabasag C.J., Bardot A., Ferlay J., Tervonen H., Shack L., Woods R.R. (2021). Colon and rectal cancer survival in seven high-income countries 2010–2014: Variation by age and stage at diagnosis (the ICBP SURVMARK-2 project). Gut.

[5] Bryant C.L.C., Lunniss P.J., Knowles C.H., Thaha M.A., Chan C.L.H. (2012). Anterior resection syndrome. Lancet Oncol..

[6] de Simone V., Persiani R., Biondi A., Litta F., Parello A., Campennì P., Orefice R., Marra A., Costa A., D’Ugo D. (2021). One-year evaluation of anorectal functionality and quality of life in patients affected by mid-to-low rectal cancer treated with transanal total mesorectal excision. Updates Surg..

[7] Croese A.D., Lonie J.M., Trollope A.F., Vangaveti V.N., Ho Y.H. (2018). A meta-analysis of the prevalence of Low Anterior Resection Syndrome and systematic review of risk factors. Int. J. Surg..

[8] Głowacka-Mrotek I., Tarkowska M., Nowikiewicz T., Jankowski M., Mackiewicz-Milewska M., Hagner W., Zegarski W. (2019). Prospective evaluation of the quality of life of patients undergoing surgery for colorectal cancer depending on the surgical technique. Int. J. Color. Dis..

[9] Sheikh-Wu S.F., Anglade D., Gattamorta K., Xiao C., Downs C.A. (2022). Positive psychology mediates the relationship between symptom frequency and quality of life among colorectal cancer survivors during acute cancer survivorship. Eur. J. Oncol. Nurs..

[10] Monastyrska E., Hagner W., Jankowski M., Głowacka I., Zegarska B., Zegarski W. (2016). Prospective assessment of the quality of life in patients treated surgically for rectal cancer with lower anterior resection and abdominoperineal resection. Eur. J. Surg. Oncol..

[11] Chen Z.H., Song X.M., Chen S.C., Li M.Z., Li X.X., Zhan W.H., He Y.L. (2012). Risk factors for adverse outcome in low rectal cancer. World J. Gastroenterol..

[12] Parks A.G., Percy J.P. (1982). Resection and sutured colo-anal anastomosis for rectal carcinoma. Br. J. Surg..

[13] Näsvall P., Dahlstrand U., Löwenmark T., Rutegård J., Gunnarsson U., Strigård K. (2017). Quality of life in patients with a permanent stoma after rectal cancer surgery. Qual. Life Res..

[14] Heald R.J., Ryall R.D. (1986). Recurrence and survival after total mesorectal excision for rectal cancer. Lancet.

[15] Cohen Z., Myers E., Langer B., Taylor B., Railton R.H., Jamieson C. (1983). Double stapling technique for low anterior resection. Dis. Colon Rectum.

[16] Law W.L., Chu K.W. (2004). Anterior resection for rectal cancer with mesorectal excision: A prospective evaluation of 622 patients. Ann. Surg..

[17] Gupta R.K., Agrawal C.S., Pathania O.P., Bajracharya A., Sah S.P., Sah P.L. (2013). Anterior resection for rectal cancer with mesorectal excision: Institutional review. Indian J. Surg..

[18] Pieniowski E.H.A., Palmer G.J., Juul T., Lagergren P., Johar A., Emmertsen K.J., Nordenvall C., Abraham-Nordling M. (2019). Low Anterior Resection Syndrome and Quality of Life after Sphincter-Sparing Rectal Cancer Surgery: A Long-term Longitudinal Follow-up. Dis. Colon Rectum.

[19] van der Valk M.J.M., van der Sande M.E., Toebes R.E., Breukink S.O., Bröker M.E.E., Doornebosch P.G., Maliko N., Neijenhuis P.A., Marinelli A.W.K.S., Peters F.P. (2020). Importance of patient reported and clinical outcomes for patients with locally advanced rectal cancer and their treating physicians. Do clinicians know what patients want?. Eur. J. Surg. Oncol..

[20] Azam F., Latif M.F., Farooq A., Tirmazy S.H., AlShahrani S., Bashir S., Bukhari N. (2019). Performance Status Assessment by Using ECOG (Eastern Cooperative Oncology Group) Score for Cancer Patients by Oncology Healthcare Professionals. Case Rep. Oncol..

[21] Weldring T., Smith S.M. (2013). Patient-Reported Outcomes (PROs) and Patient-Reported Outcome Measures (PROMs). Health Serv. Insights.

[22] Schipper S., Wiesmeth S., Wirtz M., Twork S., Kugler J. (2011). Krankheitsverarbeitungsstile und gesundheitsbezogene Lebensqualität bei Multiple-Sklerose-Erkrankten [Coping strategies and health-related quality of life in multiple sclerosis patients]. Psychother. Psychosom. Med. Psychol..

[23] O’Boyle C.A. (1992). Assessment of quality of life in surgery. Br. J. Surg..

[24] Jung W.B. (2024). Beyond survival: A comprehensive review of quality of life in rectal cancer patients. Ann. Coloproctol..

[25] Sprangers M.A.G., Cull A., Groenvold M., Bjordal K., Blazeby J., Aaronson N.K. (1998). The European Organization for Research and Treatment of Cancer approach to developing questionnaire modules: An update and overview. Qual. Life Res..

[26] Aaronson N.K., Ahmedzai S., Bergman B., Bullinger M., Cull A., Duez N.J., Filiberti A., Flechtner H., Fleishman S.B., de Haes J.C. (1993). The European Organization for Research and Treatment of Cancer QLQ-C30: A quality-of-life instrument for use in international clinical trials in oncology. J. Natl. Cancer Inst..

[27] Osoba D., Rodrigues G., Myles J., Zee B., Pater J. (1998). Interpreting the significance of changes in health-related quality-of-life scores. J. Clin. Oncol..

[28] Pape E., Pattyn P., Van Hecke A., Somers N., Van de Putte D., Ceelen W., Van Daele E., Willaert W., Geboes K., Van Nieuwenhove Y. (2021). Impact of low anterior resection syndrome (LARS) on the quality of life and treatment options of LARS–A cross sectional study. Eur. J. Oncol. Nurs..

[29] Battersby N.J., Bouliotis G., Emmertsen K.J., Juul T., Glynne-Jones R., Branagan G., Christensen P., Laurberg S., Moran B.J., UK and Danish LARS Study Groups (2018). Development and external validation of a nomogram and online tool to predict bowel dysfunction following restorative rectal cancer resection: The POLARS score. Gut.

[30] Sharp G., Findlay N., Clark D., Hong J. (2025). Systematic review of the management options available for low anterior resection syndrome (LARS). Tech. Coloproctol..

[31] Stanculea F., Ungureanu C.O., Roca D., Ginghina O., Mihailov R., Grama F., Iordache N. (2025). The impact of Low-Anterior Resection Syndrome (LARS) on the Quality of Life in Rectal Cancer Survivors: A Narrative Review. Maedica.

[32] Whistance R.N., Conroy T., Chie W., Costantini A., Sezer O., Koller M., Johnson C.D., Pilkington S.A., Arraras J., Ben-Josef E. (2009). Clinical and psychometric validation of the EORTC QLQ-CR29 questionnaire module to assess health-related quality of life in patients with colorectal cancer. Eur. J. Cancer.

[33] Lindsetmo R.O., Joh Y.G., Delaney C.P. (2008). Surgical treatment for rectal cancer: An international perspective on what the medical gastroenterologist needs to know. World J. Gastroenterol..

[34] Lupo R., Rubbi I., Barletta A., Mele C., Lezzi A., Triglia C., Botrugno I., Manca D., Potì O., Mottillo G. (2025). Quality of Life and Psychophysical Consequences in Individuals with Intestinal Stoma: An Observational Study. Int. J. Environ. Res. Public Health.

[35] Tsunoda A., Nakao K., Hiratsuka K., Tsunoda Y., Kusano M. (2007). Prospective analysis of quality of life in the first year after colorectal cancer surgery. Acta Oncol..

[36] Pappou E.P., Temple L.K., Patil S., Smith J.J., Wei I.H., Nash G.M., Guillem J.G., Widmar M., Weiser M.R., Paty P.B. (2022). Quality of life and function after rectal cancer surgery with and without sphincter preservation. Front. Oncol..

[37] Nusca S.M., Parisi A., Mercantini P., Gasparrini M., Pitasi F.A., Lacopo A., Colonna V., Stella G., Cerulli C., Grazioli E. (2021). Evaluation of a Post-Operative Rehabilitation Program in Patients Undergoing Laparoscopic Colorectal Cancer Surgery: A Pilot Study. Int. J. Environ. Res. Public Health.

[38] van der Hout A., Neijenhuijs K.I., Jansen F., van Uden-Kraan C.F., Aaronson N.K., Groenvold M., Holzner B., Terwee C.B., van de Poll-Franse L.V., Cuijpers P. (2019). Measuring health-related quality of life in colorectal cancer patients: Systematic review of measurement properties of the EORTC QLQ-CR29. Support. Care Cancer.

[39] Pachler J., Wille-Jørgensen P. (2022). Quality of life after rectal resection for cancer, with or without permanent colostomy. Cochrane Database Syst. Rev..

[40] Zewude W.C., Derese T., Suga Y., Teklewold B. (2021). Quality of Life in Patients Living with Stoma. Ethiop. J. Health Sci..

[41] Díaz-Sánchez C., Rodríguez-Muñoz P.M., Navarro-López V., Carmona-Torres J.M., Sánchez-Gil A., Sánchez-González J.L., Rivera-Picón C. (2026). Impact of Ostomy on Quality of Life in Patients with Colorectal Cancer: A Systematic Review and Meta-Analysis. Healthcare.

[42] Hudiță A., Ioana-Lavric V., Zamfir A., Buburuzan L., Ginghină O., Negrei C., Traian G., Dragomiroiu G.T.A., Costache M., Ardeleanu C. (2018). Optimization of a flow cytometry method for the approach of liquid biopsy as a therapy modulation tool in patients with colorectal cancer. Farmacia.

[43] Krouse R., Grant M., Ferrell B., Dean G., Nelson R., Chu D. (2007). Quality of life outcomes in 599 cancer and non-cancer patients with colostomies. J. Surg. Res..

[44] de Ponthaud C., Roupret M., Vernerey D., Audenet F., Brouquet A., Cotte E., Cuvillier X., Kanso F., Meurette G., Ledaguenel P. (2023). StomaCare: Quality of life impact after enhanced follow-up of ostomy patients by a home healthcare nursing service-a multicentre, randomized, controlled trial. Color. Dis..

[45] Negro S., Bergamo F., Dell’Atti L., Prete A.A., Galuppo S., Scarpa M., Bao Q.R., Ferrari S., Lonardi S., Spolverato G. (2025). Quality of Life in Rectal Cancer Treatments: An Updated Systematic Review of Randomized Controlled Trials (2013–2023). Cancers.

[46] Fernández-Martínez D., Rodríguez-Infante A., Otero-Díez J.L., Baldonedo-Cernuda R.F., Mosteiro-Díaz M.P., García-Flórez L.J. (2020). Is my life going to change?—A review of quality of life after rectal resection. J. Gastrointest. Oncol..

[47] Manocchio N., Vita G., Giordani L., Ljoka C., Monello C., Foti C. (2025). Rehabilitation for Women and Men Experiencing Sexual Dysfunction After Abdominal or Pelvic Surgery. Surgeries.

[48] Milbury K., Cohen L., Jenkins R., Skibber J.M., Schover L.R. (2013). The association between psychosocial and medical factors with long-term sexual dysfunction after treatment for colorectal cancer. Support. Care Cancer.

[49] Zigouras S., Taylor J.P. (2025). Sexual Dysfunction after Colorectal Surgery. Clin. Colon Rectal Surg..

[50] Lauwereins L., D’Hoore A., Coeckelberghs E., Fieuws S., Wolthuis A., Bislenghi G., Van Molhem Y., Van Geluwe B., Debrun L., Devoogdt N. (2025). A 2-year prospective study on the evolution of Low Anterior Resection Syndrome (LARS) following rectal cancer surgery. Int. J. Color. Dis..

[51] Sun R., Dai Z., Zhang Y., Lu J., Zhang Y., Xiao Y. (2021). The incidence and risk factors of low anterior resection syndrome (LARS) after sphincter-preserving surgery of rectal cancer: A systematic review and meta-analysis. Support. Care Cancer.

[52] He S., Zhang J., Wang R., Li L., Shi L., Ren D., Wang J., Deng Y., Dou R. (2022). Impact of long-course neoadjuvant radiation on postoperative low anterior resection syndrome and stoma status in rectal cancer: Long-term functional follow-up of a randomized clinical trial. BJS Open.

[53] Zhang X., Meng Q., Du J., Tian Z., Li Y., Yu B., Niu W. (2024). Risk factors of the low anterior resection syndrome (LARS) after ileostomy reversal in rectal cancer patient. Sci. Rep..

[54] Slørdahl K.S., Balto A., Guren M.G., Wibe A., Kørner H., Norderval S., Gjelsvik Y.M., Myklebust T.Å., Larsen I.K. (2024). Patient-reported outcomes after treatment for rectal cancer-A prospective nationwide study. Color. Dis..

