# Supplementary material for: Quality of Life and Functional Outcomes After Rectal Cancer Surgery: A Comparative Study Applying EORTC QLQ-C30, QLQ-CR29, and LARS Score at 1–6 Months Postoperatively
Source: Healthcare (Basel). 2026 Apr 30;14(9):1203. doi: 10.3390/healthcare14091203 (PMC13163284; doi:10.3390/healthcare14091203)
Supplement: Supplementary file 1 [file healthcare-14-01203-s001.zip › File S1 EORTC QLQ-CR29.pdf]

EORTC QLQ – CR29

Pacienţii semnalează uneori următoarele simptome sau probleme. Vă rugăm să indicaţi gradul în care aţi prezentat aceste simptome sau probleme în ultimele 7 (şapte) zile. Vă rugăm să răspundeţi încercuind numărul care se potriveşte cel mai bine situaţiei dumneavoastră.

**În ultimele 7 (şapte) zile:**	**Deloc**	**Puţin**	**Destul de mult**	**Foarte mult**
31. Aţi urinat des în timpul zilei?	1	2	3	4
32. Aţi urinat des în timpul nopţii?	1	2	3	4
33. Aţi avut pierderi (scăpări) involuntare de urină?	1	2	3	4
34. Aţi avut dureri la urinare?	1	2	3	4
35. Aţi avut dureri abdominale?	1	2	3	4
36. Aţi avut dureri ale feselor/zonei anale/rectului?	1	2	3	4
37. Aţi avut senzaţia de balonare a abdomenului?	1	2	3	4
38. Aţi avut scaun cu sânge?	1	2	3	4
39. Aţi avut scaun cu mucus?	1	2	3	4
40. Aţi avut gura uscată?	1	2	3	4
41. Aţi pierdut păr în urma tratamentului?	1	2	3	4
42. Aţi avut probleme cu simţul gustului?	1	2	3	4

**În ultimele 7 (şapte) zile:**	**Deloc**	**Puţin**	**Destul de mult**	**Foarte mult**
43. V-aţi făcut griji pentru sănătatea dumneavoastră în viitor?	1	2	3	4
44. V-aţi făcut griji în legătură cu greutatea dumneavoastră?	1	2	3	4
45. V-aţi simţit mai puţin atractiv/ă din punct de vedere fizic ca urmare a bolii sau tratamentului dumneavoastră?	1	2	3	4
46. V-aţi simţit mai puţin feminină/masculin ca urmare a bolii sau a tratamentului dumneavoastră?	1	2	3	4
47. Aţi fost nemulţumit/ă de corpul dumneavoastră?	1	2	3	4
48. Aveţi pungă colectoare (colostomă/ileostomă)? (vă rugăm să încercuiți răspunsul corect)	Da		Nu	

Vă rugăm să continuaţi cu următoarea pagină

În ultimele 7 (şapte) zile:

	Deloc	Puţin	Destul de mult	Foarte mult
**Răspundeți la aceste întrebări DOAR DACĂ AVEȚI STOMĂ (PUNGĂ COLECTOARE); dacă nu, vă rugăm să continuați mai jos:**				
49. Ați avut scăpare involuntară de gaze/flatulență din stomă (pungă colectoare)?	1	2	3	4
50. Ați avut scurgeri de scaun din stomă (pungă colectoare)?	1	2	3	4
51. Ați prezentat durere a pielii în jurul stomei?	1	2	3	4
52. Au avut loc schimbări frecvente ale pungii colectoare peste zi?	1	2	3	4
53. Au avut loc schimbări frecvente ale pungii colectoare peste noapte?	1	2	3	4
54. V-aţi simţit jenat/ă din cauza stomei?	1	2	3	4
55. Ați avut probleme cu îngrijirea stomei?	1	2	3	4

**Răspundeți la aceste întrebări DOAR DACĂ NU AVEȚI STOMĂ (PUNGĂ COLECTOARE):**				
49. Ați avut scăpare involuntară de gaze/flatulență din anus?	1	2	3	4
50. Ați avut scurgeri de scaun din anus?	1	2	3	4
51. Ați prezentat durere a pielii în jurul zonei anale?	1	2	3	4
52. S-a întâmplat peste zi să aveți un tranzit intestinal accelerat?	1	2	3	4
53. S-a întâmplat peste noapte să aveți un tranzit intestinal accelerat?	1	2	3	4
54. V-aţi simţit jenat/ă din cauza tranzitului intestinal?	1	2	3	4

În ultimele 4 (patru) săptămâni:

	Deloc	Puţin	Destul de mult	Foarte mult
**Doar pentru bărbați:**				
56. Cât de interesat aţi fost de sex?	1	2	3	4
57. Aţi întâmpinat probleme la obţinerea sau menţinerea erecţiei?	1	2	3	4

**Doar pentru femei:**				
58. Cât de interesată aţi fost de sex?	1	2	3	4
59. Aţi avut dureri sau disconfort în timpul contactului?	1	2	3	4

© QLQ-CR29 Drepturi de autor 2006 EORTC Quality of Life Group. Toate drepturile rezervate. Versiunea 2,1
