# Supplementary material for: Quality of Life and Functional Outcomes After Rectal Cancer Surgery: A Comparative Study Applying EORTC QLQ-C30, QLQ-CR29, and LARS Score at 1–6 Months Postoperatively
Source: Healthcare (Basel). 2026 Apr 30;14(9):1203. doi: 10.3390/healthcare14091203 (PMC13163284; doi:10.3390/healthcare14091203)
Supplement: Supplementary file 1 [file healthcare-14-01203-s001.zip › File S2 SCORE LARS.pdf]

ANEXA 2

SCORE LARS

Instrucțiuni de completare

Acest chestionar are scopul de a vă evalua funcția digestivă în urma intervenției chirurgicale. Vă rugăm să marcați o singură căsuță la fiecare întrebare. Se poate să fie dificil să selectați un singur răspuns având în vedere că pentru unii pacienți simptomele sunt diferite de la zi la zi. Vă rugam să selectați răspunsul în care vă regăsiți cel mai bine. Dacă ați avut de curând o infecție care v-a afectat funcția digestivă, vă rugăm să nu o luați în considerare. Vă rugăm săalegeți răspunsul carevă descrie funcția digestivă zilnicăîn condiții normale.

Întrebări

1. Există ocazii în care nu vă puteți controla flatulența (vânturile)?		
a) Niciodată	☐☐	0
b) Da, mai puțin de o dată pe săptămână	☐	4
c) Da, cel puțin o dată pe săptămână		7
2. Există ocazii în care aveți scaune lichide necontrolate/accidentale?	☐☐	
a) Niciodată	☐	0
b) Da, mai puțin de o dată pe săptămână		3
c) Da, cel puțin o dată pe săptămână		3
	☐☐	
3. Câte scaune aveți pe zi?	☐	
a) Mai mult de 7 ori pe zi (24 de ore)	☐	4
b) Între 4-7 ori pe zi (24 de ore)		2
c) Între 1-3 ori pe zi (24 de ore)		0
d) Mai puțin de o dată pe zi (24 de ore)		5
	☐☐	
4. Se întâmplă să aveți scaun la mai puțin de o oră de la ultimul scaun?	☐	
a) Niciodată		
b) Da, mai puțin de o dată pe săptămână		0
c) Da, cel puțin o dată pe săptămână		9
	☐☐	11
5. Aveti vreodată o nevoie atât de puternică de a avea scaun încât să fiți nevoit sa vă grăbiți la toaletă?	☐	
a) Niciodată		
b) Da, mai puțin de o dată pe săptămână		0
c) Da, cel puțin o dată pe săptămână		11
		16

Rezultat

Data:_______________ Punctaj total:_______

☐ 0-20 No LARS

☐ 21-29 Minor LARS

☐ 30-42 Major LARS
